# Thoughts on Medical Research

**Published:** 1853-12

**Authors:** 


### ART. IV.—*Thoughts on Medical Research.*

By Rusticus.

Messrs. Editors,—Sitting here in my little office and listening to the beating of the storm without, my mind has wandered somewhat from the every-day details of practice, to those fountains whence our skill proceeds.

Looking at my scanty library as it stands in the dim light of a single lamp, I have been drawn back from the present to the past, and have looked beyond and within the leathern coverings of my books, to the toilsome thoughts, and weary headaches, which gave them birth.

They are old books; some of them running back two hundred years, printed in black letter, and written in the sonorous Latin of the mediæval literature. Those books are relics, grim mementoes of the times when Lower and Musgrave, and Turberville, and Tyson, and Wren, and Fairfax, and Harvey, lived and toiled in England; while on the continent Malpighi, Lancisi, and Von Helmont joined the eager chase for fame. There I read of the "Incomparable Malpighi, who industriously applied himself to very serious Studies, was of a good Habit of Body, and had seen 66 Years; but he had frequent Sicknesses; sharp Vomitings did torment him for 20 Years; he was troubled with the Gravel, a Hæmorrhagia in the Kidneys, a Rheumatism Fluxious; which with their troublesome Consequences augmented his Infirmities. Scarce had these Evils given him some respite, when a cruel Palpitation of the Heart, with an unequal Pulse came upon him. Moreover, 4 Years before his Death, a sharp and biting Sweat failed not, all the Summer to trouble him every Night. Pope Innocent XII. having call'd him to Rome to make him his Chief Physician, he began the first Year to lose his fresh Colour; in the second he voided many Stones without much Pain; and in the third, which was the last of his Life, he found himself oppress'd during Winter with a Difficulty of Breathing. His Health being thus insensibly undermined, and a Bilious Looseness returning ever and anon, he was at length seized with a Vertigo, a Loss of Speech, and a Contortion of the Mouth, (*Spasmus Cynicus,*) and a Palsy of the Right-side. And tho' there was appearance that he was out of danger by Bleedings, Purges, Diureticks, and Antapoplectick Medicines, yet one might see, by his Melancholy Countenance, but especially his want of Memory, that there was lodg'd in his Brain some Melancholy Humour. Therefore perceiving his End drawing near, he sign'd with his Hand 3 Days before his Death, his Posthumous Works, which he had order'd to be deliver'd to his Collegues of the *R. Society at London.*

"Then having confess'd himself with great Humility, he attended generously, and with Faith in GOD, the Death which appear'd to him certain, and not far off. And on the 28th of *Nov.*, 1696, a terrible *Apoplexy* finish'd, in the Space of 4 Hours, this so precious Life."

Truly was this a precious life—laborious under suffering, and faithful under affliction. In the day when he lived there were giants. The Harveian discovery had opened the way for a research illimitable, and unending. Anatomy was for the first time a science; in their dissections those grave old men saw wonders, such as the modern dissector cannot see; the scalpel in their hands was as the prow of a ship, clearing its way to unknown countries; and the discovery of a new organ came to the sense of the anatomist,

> "welcome as the cry
> That told the Indian isles were nigh
> To the world-seeking Genoese,
> When the land-wind, from woods of palm,
> And orange-groves, and fields of balm,
> Blew o'er the Haytian seas."

It is one of the pleasant things in practical anatomy, to look upon the Malpighian pyramids and corpuscles, and think back almost two centuries to their first description. It is by this nomencature that Malpighi lives in science to the present day. So also Wharton, and Steno, Ingratius, and Vieussens, all send their names down to posterity.

But the drowsy beating of the rain has rendered me discursive. I do not write a reverie. Well—these old men lived, and wrote, and died, and I have their souls in leathern covers, within which I look, and hold converse at mine ease. O shade of Malpighi! When first you told us of the pyramids, dreamed you of the dark decades which should pass before we knew their office? Thought you that now, here in the nineteenth century, you living in the seventeenth, should equal us in knowledge?

Now O Editors! you have my text. Medical research is a snail, a very turtle in progression. The march of centuries finds us still in the maze of hypothesis. Death sits like the Minotaur within the Labyrinth of Crete, and his victims reach by various mazes, but with unvarying certainty their inevitable doom.

So it is that we sit in sadness, and behold the fearful epidemic sweeping the wide world in relentless wrath. We witness, year after year, the same diseases presenting the same unchanging routine of symptoms, described but not known, treated but not cured, combated but not prevented. A sphynx-like gloom involves the subject of causation, of propagation, of nature, and of treatment. How shall we solve this riddle, how enlighten the darkness which Harvey's discovery made visible?

Two routes are before us. Vesalius and the older physicians studied and experimented as to facts. Out of this previous knowledge, and the consideration of final causes, came the Harveian discovery of the circulation. Then came the cry *Eureka!* To the dazzled eyes of Von Helmont, and Musgrave, and Lower, it seemed that the premises were all laid down, the quantities and their conditions all stated, and the problem to be solved by logical analysis.

Of all the misty errors, of all the profound obscurities, of all the learned follies that ever disgraced the annals of science, none were so absurd as those of logical medicine. Men left the dissection room and laboratory, and sat quietly down in their studies to *reason* out a treatment of disease. Starting without facts, and *with* falsehoods, they built upon their blunders a series of theoretical magnificences, as grand, as obscure, unmeaning and useless, but not as permanent, as the pyramids. Theirs were great minds. They were the profoundest of students, but where are the results of all this greatness and profundity? Turn, mine Editors, to the history of Malpighi's illness, and read in that brief record the failure of their skill, the emptiness of their knowledge.

Now ye who write so learnedly—ye who lecture unto classes—ye great minds, and profound students of modern medicine—tell us from the long catalogue of symptoms, what was the disease which slew Malpighi, and how you would have cured it. Have you in all your dictionaries words which tell more than the quaint phrases of that pathetic record? He had a "melancholy humour in his brain." You laugh at the oddity of the expression; you may deny its truth, but can you do more? Can you tell us what he had in his brain—what was its mode of action—what was its causation? Alas, the whole learning of modern science is insufficient to the answer.

And why? Because we have stopped at pathological appearances. The revelations of the microscope, and the searching of chemical analysis, have been neglected. The eye alone is incapable to the appreciation of anything more than the effects of a cause—the cause itself is left untouched. Call in the microscope, and you may see visible changes in the blood, or in the texture of its vessels. Or failing that, the *experimentum crucis* may reveal irregularities in its component parts, a loss of definite proportions, or the presence of abnormal elements.

These are your data. From them you may reason to causation, and from causation to cure. Without them all theoretical notions will be as vague, and monstrous in absurdity, as those by which Lancisi accounted for the death of his friend and colleague, Malpighi. Aside from the quaintness of the old translation, and the change of nomenclature, I have seen in the medical periodical press of the past year, a score of mis-named theories, as probable as this which I now quote:

"The *Right Ventricle* of the *Brain* contain'd almost two Ounces of extravasated Blood, and the *Left Ventricle* was swell'd with a thick and yellow sort of Phlegm, which weighed more than an Ounce. Moreover, the *Dura Mater* stuck closer to the *Scull* than is usual. This proves,* that the *Conglobated* Glands in the whole Body had thrown into the Mass of Blood an Acid Lymph, and that the *Conglomerated* Glands of the Hypochondria, especially those of the Liver, had thrown into it a Melancholy Humour, and that these two Sorts of Humours, being carried into the Vessels of the *Brain*, had dispos'd the Blood to coagulate there, and that these having corroded and broken the *Tunicles*, which serv'd for a stop to them, they had run into the Cavities, where they caus'd Death without a Remedy."

I presume, good Editors, that you don't understand this rationale. Turn, then, to the latest author on cerebral disease, and see whether he can tell you more of the intimate nature of apoplexy. He will not ascribe the rupture of the coats of the artery to an acid erosion, but will stop at the very fact of erosion, or frangibility from albuminous and ossific deposit, and will leave you at last as much in the dark as to ultimate causation as ever. Possibly Lancisi is the nearer right. Undoubtedly he is right in assigning the early causation to the *primæ viæ.*

Is this hypothesis of Lancisi absurd? Pause before you decide. Lancisi was a great man. Two hundred years ago, he told us all that we now know of malaria and marsh miasm. He then demonstrated the causation of the periodical fevers, and drew from the Pontine marshes a true theory, which has stood the test of centuries. He proved too much, it is true, but was he the last discoverer who has mingled great truths with somewhat of error?

As an instance of those strange insanities which beset the originator or follower of hypotheses, suffer me to make some brief extracts from an article full of that shrewd reasoning which always marks the advocate of error — an article published during Anno Domini, 1853, in a very respectable western medical monthly, and that without a word of negation or remonstrance from the editor, whose name is one deservedly reputable on both sides the Atlantic. Arguing the causation of dysentery, thus speaks our *Lancisi redivivus:*

"Its wayward and whimsical mode of progression is consistent alone with the fickleness of insect migration. * * * * * The first discoverable symptom, is that which might arise from the stimulation produced by the titillation of the mucous membrane by the little intruder, on first taking possession of the part, viz., an increase of the natural secretion, but presently

---

* O most admirable of proofs, most logical of reasonings!

they make an entrance into the substance of the membrane, destroying its texture, and exposing the open mouths of the capillaries, irritate the nervous extremities with which the part is so abundantly supplied, and of course get up a high state of morbid excitement"— which, gentle reader, is dysentery.

Now, from the consideration of this causation of dysentery, our author reasons, *very logically*, that something which will destroy insect life, is the true remedy for dysentery. Bed-bug Bane is a little too active. Lyon's Magnetic Pills and Powders are within the domain of quackery—but our author has his resources. He has noticed that peach-tree bark and sassafras are fatal to lice, on "puppies, and the heads of little negroes." Hence the following "clincher" to his argument:

"It is therefore evident that the disease of the bowel in flux, is of a specific nature, gotten up by the agency of a specific cause, and that that cause is the presence of poisonous animalculæ, is rendered still more probable by the fact that those means known to be most destructive to insect life, are most effectual in arresting this disease. This is eminently true with regard to the peach-tree and sassafras."

You will urge that I am quoting an extreme case of medical theorizing. But extreme cases are only the results of milder errors, and serve the purpose of my illustration. What I mean to din into your ears, and into the hearts of medical reasoners, is that medical reasoning is the same now as in the days of Lancisi, and must inevitably (no matter how acute the mind, or clear the head that argues) result in error.

Humiliating confession! Dark evidence of weakness and of ignorance! All reasoning in medicine must fail for want of data. The labor of so many years, the recorded experience of so many toilsome lives, does not yet suffice for the construction of a medical theory. I am weary. For years I have watched disease wherever

"Mors æquo pede pulsat turres regum
Et tabernas pauperum."

Is there no end to my task? Somewhere, deep hid in nature, lie the facts we seek, and they alone can help us. Reason is a Will o' the Wisp, ever leading us astray. Logic and metaphysics are humbugs. In facts and their analysis lies our only safety. I would say to the profession, as did old Horace to the ship of state:

"O navis, referent in mare te novi
Fluctus! O quid agis? Fortiter occupa
Portum."

Let us come back to facts—let us carefully collect and analyze them, and as you, O Smelfungus, once said, we shall anon, as we rise from the trough of the sea, behold the load star of certainty in medicine.*

I have written discursively. My style, as well as my argument, may go to prove that I am unfit for a medical reasoner. I trust I am. Much as I love old books, and fascinating as are the misty theories thrown about disease, I leave them in my office when I go to the bedside. In the presence of disease I see the futility of theory, and turn to the simple facts of some clinical report, as to a friend in need. In earlier life, when some ambitious hopes of being at some time a medical philosopher possessed me, I lost some patients by my devotion to creeds. With a decreasing faith in medication, I have an increased veneration for medical science, and its usefulness.

But the night wears on apace. My lamp grows dim, and the old books on the shelves loom out so darkly, that I can (as one who gazes long on living coals sees cities there) behold the fathers of medicine peering out solemnly from among them, with faces sad and mournful. I shall leave them now, and on some other rainy night I may talk more of all these things. Adieu.

* Our correspondent gives Smelfungus more than his due. The beautiful metaphor he quotes, may be found in Carlyle's "Past and Present."—Editor.

